# “I am so happy this community exits”: an analysis of the benefits and harms of dementia self‐disclosure on social media

**DOI:** 10.1002/alz70858_099145

**Published:** 2025-12-24

**Authors:** Mallorie T Tam, Angela Peng, Mahala G English, Viorica Hrincu, Zijian An, Kenneth A Joseph, Julie M. Robillard

**Affiliations:** ^1^ University of British Columbia, Vancouver, BC, Canada; ^2^ University at Buffalo, Buffalo, NY, USA; ^3^ University of Buffalo, Buffalo, NY, USA

## Abstract

**Background:**

An increasing number of people living with dementia and their care partners are turning to social media platforms to seek advice, access information, share personal stories, and connect with others for support. This engagement often involves the disclosure of a dementia diagnosis or identification as a care partner, a practice that carries both potential risks and benefits. Social media use has been shown to influence mental and physical health, foster community connections, and influence patient‐caregiver dynamics. However, significant concerns remain regarding the potential risk of exposure to misinformation and stigma as a result of self‐disclosure. The current project aims to explore the underlying motivations and potential impact of self‐disclosure on social media.

**Method:**

We performed a content and thematic analysis of public social media posts obtained from Facebook and Reddit. A total of 22,101 posts related to self‐disclosure were retrieved from 36 Facebook groups and pages over a six‐month period. Of these, a sample of 1,621 posts were selected for final coding and thematic analysis. Additionally, a total of 1,032 posts were collected from Reddit, with a final sample of 779 posts retained after applying exclusion criteria. To gain deeper insights into exchanges surrounding dementia care, comments and replies to 153 Facebook posts and 187 Reddit posts were also analyzed.

**Result:**

Preliminary findings indicate advice‐ and information‐seeking as the predominant motivations for self‐disclosure, with users frequently requesting personal experiences and insights into the complex symptomology. A response analysis revealed that the majority of Facebook users either provided information, including advice, or related to the original poster's circumstances. Initial thematic analysis identified key themes, including complex and iterative grief, challenges associated with end‐of‐life decisions, and the compounding nature of functional decline.

**Conclusion:**

The insights uncovered in this work will guide the development of an evidence‐based resource to support decision‐making around social media use in dementia.

